# Selections from Foreign Journals

**Published:** 1888-01

**Authors:** 


### SELECTIONS FROM FOREIGN JOURNALS.

Original Investigation on the Ætiology of Tetanus.—By Dr. A. Bonome, First Assistant in the Anat-Pathol Institute of the University of Turin ; *Fortscritte der Medicine*, November, 1887.

The experimental and bacteriological results obtained recently in the investigation of the ætiology of tetanus, have not only established its exact nature, but also from the standpoint of the pathology of human tetanus, an absolute certainty as to the character of the disease.

The first positive information previous to November, 1886, was given by Rosenbach, who produced tetanus in two guinea pigs by the direct subcutaneous inoculation of a small clipping from a carbuncle of a tetanus patient. He established the existence of a definite bristle-shaped bacillus, in which there were no end spores. It was identical with that found by Nicolaier in earth. This isolated case upon which the pathogenesis of human tetanus was based, was in November confirmed by me, and later by Hochings, Vanni and Garre.

During the year I was able to make several observations on men, horses and wether rams. The three cases of human tetanus which I saw were caused by some wounds received by the ruins of a church, in February, 1887. In these cases in the stinking secretions of the necrotic wounds, I found the bristle-shaped bacillus together with a mass of micrococci, bacteria and bacilli of various forms and sizes. This bristle-shaped bacillus was easily stained with fuchsia and was two or three times as long, thicker and straighter than the tuburcle bacillus. The extremities presented a swollen point, giving it the appearance of a pin. In the blood, in the cerebro-spinal fluid, in the substance of the nerve centers, in the nerve juice, and in the intestines these bacilli were absent, and inoculations with material from these parts gave no result. Inoculations with the pus secretions from the tissue in the neighborhood of the wounds, on the other hand, produced tetanus in animals inoculated subcutaneously, or in the substance of a muscle. In the exudate from the inoculated part, I was always able to find the bristle and the pin-shaped bacillus. Some of the animals inoculated died suddenly of general tetanus before any exudate appeared. In these cases cultures made from the juice of the very hyperæmic muscle tissue, showed the above spoken of bacilli, although they were not to be found in the muscle tissue about the place of inoculation. Subcutaneous inoculations of this hyperæmic muscle tissue produced tetanus in animals.

The bacilli produced tetanus after having been dried for four months.

Inoculations made in blood serum, gelatine and peptonized agar-agar, caused the materials to become liquid in the upper portion. This liquified mass contained besides micrococci, three different sorts of bacilli, the one short and thick, rounded at the end, the second thinner and longer, and at the same time feather-shaped, while the third was even thinner but shorter, and often with a spore at the end and plainly colored in the center. In this liquid portion I seldom found the bristle and pin-shaped bacillus, but it was always present in the shrunken solid portion at the bottom of the tube, where complete decomposition had not taken place. Only very seldom could I produce tetanus by the inoculation of the liquid part of the culture.

Temperature experiments were made by drying culture of from five to ten days incubation, on sterilized watch glasses and submitting them to a temperature of 30° C. in the thermostat for from fourteen days to four months. Cultures were then made according to the anærobic method, an animal immediately inoculated and cover glass suppurations examined. There were present two sorts of bacilli and a large micrococcus together with the bristle-shaped bacillus. Inoculations of these in sterilized water and submitted to a temperature of 55° to 60° for ten minutes, the large micrococci were destroyed. Again submitted to a temperature of from 55° to 80° and the three sorts of bacilli remained alive. The bristle-shaped, the short with spores, and the larger feather-shaped bacillus without spores. Finally, I found that continuous heating for five minutes at a temperature 100° destroyed the feather-shaped bacillus. Inoculations made with these two remaining organisms did not liquify the blood serum cultures. It was observed, moreover, that by the separation of the discomposition bacillus from the cultures the growth of the bristle-shaped organism was greatly retarded. From time to time attempts were made to produce tetanus with the pure cultures. Liquified gelatine cultures containing only the decomposition bacillus, moreover, never produced tetanus in animals inoculated. Both together always produced the disease.

It must be, then, that tetanus in animals as in man is produced by an agent which is a fine bristle, and pin-shaped bacillus identical with that found by Nicolaier and that the decomposition bacillus has no tetanus-producing qualities. There exists, however, a true companionship between these two organisms and they are naturally bound together.

The tetanus bacilli have been found in the lime dust of old buildings and inoculations of such material when the bacilli were present produced the disease in animals. In one instance several persons were injured by the falling of an old building, and of the number injured several died of tetanus. The lime dust debris contained the bacilli as also did the exudations from the necrotic wounds. Animals inoculated with both materials died of tetanus.

In another instance several people were injured in a similar manner but none died of tetanus, and neither was the organism found in the exudate from the wounds, nor in the lime dust debris. More over, the disease could not be produced in animals inoculated with this lime dust or with the exudation.

A horse in which an open wound produced by a fall became filled with street dust died three days later with tetanus. The characteristic bacilli were present in the exudate and animals inoculated died of the disease.

In a ram which had been castrated, tetanus followed the operation, and I was able, while the animal was living, to obtain the bacillus from necrotic wounds of the scrotum, and to produce the disease in animals inoculated with this material.

It was not determined what degree of heat was necessary for the destruction of the tetanus bacillus, or how long it could withstand the drying process.

A. W. Clement, V. S.

Salicylate of Soda in Rheumatic Affections in the Horse.—M. Condamine (*Rec. de Med. Vet. Nov.* 1887), offers the following conclusions on the use of the above salt, as the result of a series of experiments and careful analyses:

"If the Salicylate of Soda as a curative agent does not act with certainty on all subjects, and in all forms of rheumatism, it at least has an effect on the pain, which it attenuates or drives rapidly away. From the outset it lowers the temperature and consequently abates the fever. It favors the transformation of albuminoids and other nitrogenous principles of the blood, which are eliminated from the economy by the diuretic action of the salt. The combined actions of this remedy, act in a favorable manner on rheumatic affections, and abridge considerably the course of the disease."

Effect of Salting on the Virulence of Anthrax Meat.—Prof. Peuch, of Toulouse, states that when the salting of pork which has been affected with Anthrax is complete, the juice extracted by pressure has lost all virulence, which is explained by the fact that in the meat as in the cadaver, the bacteridia remains in its filamentous state. If on the contrary, the salting has been incomplete, the deeper layers have preserved their virulence.

Unilateral Pleuresy in the Horse.—M. Barrier ( *Rec. de Med. Vet. Ang.* 1887). Eighteen months ago no one believed in the existence of unilateral pleuresy in the horse. Those who found it in autopsies or diagnosed it on the living animal, had not risked the publication of their observations, so classic is the opinion that double pleuresy is the only possible condition.

If the perforation of the mediastinal septum is the rule it is far from being the law. It is subject to numerous exceptions. "The posterior mediastinum of the horse is a membrane absolutely imperforate in certain cases, during life and after death."

It is generally excessively delicate, which renders it subject to perforation, during life or after death, from very slight causes. The perforations have the character of recent endothelial tears, and not to the analogous natural openings of the great omentum.

The openings described at the autopsy as the normal condition are *probably* only partial ruptures, produced accidentally by the manipulation of dissection. *There can exist* in the horse, *unilateral* inflamatory exudations of the pleura, *momentary* when the septum is delicate, or permanent when it is firmer.

Pleuresy is excessively difficult if not impossible to detect by percussion at the very outset, as it is at this moment probably always unilateral, and the exudation is only recognized when it has passed the line of dullness of the lower part of the thorax.

Lastly, if the normal formability of the medastinum explains the ordinary bilateral pleuresy in the horse, it does not exclude the unilateral condition at the commencement.

In clinical support of his anatomical discovery M. Barrier publishes the clinical history and autopsies of a number of cases of unilateral pleuresy.

R. S. HUIDEKOPER, *Veterinarian.*

